# Case of Severe Wound of the Brain

**Published:** 1871-11

**Authors:** Wm. Abram Love

**Affiliations:** Atlanta, Georgia


### CASE OF SEVERE WOUND OF THE BRAIN.

*Loss of two Fluid-Ounces of Brain Substance, a Large Amount of Bony Covering, and Recovery without Impairment of Mind.*

BY WM. ABRAM LOVE, M.D., ATLANTA, GEORGIA.

In the afternoon of February 24th, 1868, I was called, with Dr. W. L. Davis, to see Yuel C., age fourteen years, son of Captain Y. G. Rust, Albany, Georgia, who had been very severely wounded while on his way from school, only a short time before.

Found him lying on his back—head elevated—covered with blood, which was still oozing from a severe wound in the head, inflicted with a common axe in the hands of a negro. He recognized me on entering the room, but an effort to speak brought on a second severe convulsion, one having preceded our arrival. His pulse was quick, weak, and somewhat irregular. The convulsions arrested any further immediate examination. Chloroform was given him immediately, which served to control the convulsions very readily, and when anæsthised, we proceeded to examine the nature and extent of the injury, while we at the same time learned from the family and friends a few of the particulars of the case, which may not be out of place here.

On returning from school, he was attacked by a negro, who struck him with the blade of an axe, the two parties facing each other at the time. The blade entered over the left superciliary ridge, a little to the left of the axis of left eye, cutting inward, downward and backward, inflicting a wound five inches in length through the scalp, penetrating through the skull, membranes and brain, a like distance, burying the blade deep into the brain and near the centre of the left cerebral hemisphere, in the direction of the left occipital foramen; then the axe, describing a curve, the mid-portion of the blade resting on the right cut edge of the os-frontis as a fulcrum, split off the left side down to the squamous suture, dashing out that portion of the brain, leaving the head laid open five inches. The patient fell, but soon rallied, and, with the assistance of his schoolmates, walked home, (some two or three hundred yards), bleeding, faint, weak, and occasionally falling, but conscious. He requested his parents (who were absent) and the doctors sent for. After this he was taken with a convulsion, and at the onset of the second paroxysm we had just reached the room.

A hurried examination showed the bones broken into several pieces, each entirely detached from the other—the cerebral substance broken up and thrown out into the hair, on the face and clothing, and the blood still flowing from some invisible vessel.

No time was lost in dissecting the fragments, which was done by Dr. Davis.

Hoping that, in the event of recovery, the *periosteum and dura mater* might ultimately develop a new bony covering, the utmost care was taken to preserve, in contact with the scalp, every particle of the periosteum. Just when the apparently last of the fragments of bone were detached and removed about two and a half by three and a half inches, another convulsion came on, notwithstanding the patient was still under the influence of chloroform to insensibility. The anæsthetic was increased by Dr. Conally, who was present, and other fragments of bone searched for in the broken-down cerebral mass. Buried about two inches and a half into the brain substance, just below the upper back part of the wound, near the centre of the cerebrum, was discovered another fragment of bone, which, on removal, was found to be a piece of the inner table, of irregular diamond shape, measuring an inch by an inch and three-quarters. This had been drawn down by the dull blunt corner of the axe.*

When this last fragment of bone was removed, the convulsions ceased, not to return again. The edges of the bones were smoothed, the coagula and the broken-down brain-tissue carefully removed. A small inter osseal artery, which still continued to bleed, gave some trouble, it having retracted into the bony canal. This was ultimately controlled by pressure, when the wound was left open until the effects of the anæsthetic had passed and reaction taken place. No hemorrhage occurring then, the wound was closed with silver pins and figure 8 sutures, supported between by adhesive strips—a dose of brandy and morphine given—the patient washed, cleanly clad, and put to bed under cold-water dressing.

During the night he slept at longer or shorter intervals, arousing occasionally and calling for water, which was given at pleasure; ice-water dressing continued to the head. Pulse and respiration continued good throughout the night, with little mental disturbance evinced at any time. Kidneys acting well.

25th.—Small quantities of sanguineous fluid discharged from wound at lower point over superciliary ridge; otherwise lips adherent by plastic lymph. Pulse one hundred and twenty, soft, weak; respiration normal; mind clear. Toward evening, slight febrile excitement. Gave laxative to unload

---

*The axe had been broken at this corner and then ground down to an edge, but was thick and dull; hence, this piece of the internal plate and a small portion of the external plate had not been *cut* but *broken in* and driven before this portion of the blade by the force of the blow.

bowels—bland fluids to aid; ice and ice-water internally and externally. Bowels moved during the night.

26th, A.M.—Has passed the night comfortably. Some œdema over left eye and along wound in scalp, with tendency to extend itself. Fearing erysipelas, gave tinct. ferri chlor., and continued local applications. He expressed a desire for fish.

P.M.—Severe rigors, followed by febrile excitement, with delirium. Gave ice freely, with ice-water to head, and continued iron.

27th.—During night fever passed, with diaphoresis giving evidence of malaria, which was also indicated by the condition of the tongue. Has slept "short naps" during night. Œdema of face and scalp on the increase; pulse one hundred and twenty, weak; otherwise comfortable. Has taken a few oysters and a little weak egg-nogg, but desires fresh fish. No exudation from wound during the day; one of the sutures disposed to give way from the swelling. Continued treatment, adding tinct. nucis vom. and liq. pot. arsenit. to the tinct. ferri chlor. It is noticed, that during febrile excitement the scalp protudes, where the bony covering has been removed, into a pulsating "tumor," which subsides again on the exacerbation of the fever.

28th.—Wound discharging freely; surface healthy in appearance; œdema of face and scalp not increased. Has passed the night comfortably. Bowels moved; kidneys and skin acting; mind clear, with a very great craving for fish. At four o'clock P.M. a distinct chill came up, followed by fever, which passed off during the night, with free perspiration. The tongue giving additional evidence of malarial disturbance, on inquiry, it was ascertained that he had for some time been suffering from chronic chills and fever. The tinct. iron disagreeing with the stomach, he was directed ferri protocarb., ext. nucis vom., ext. hyociam. and acid arsenious, in pill form *ter dies*, to break up the malarial disturbance, fearing the quinine in the injured condition of the brain. Fresh fish were allowed him when they could be obtained, and in their absence, sardines—the only articles of food he seemed at all to relish, and for these he had a most inordinate craving; henceforward they constituted his standing bill of fare.

29th.—During the night he evinced more or less delirium, with incoherent muttering when asleep. Bowels have been moved. Skin and kidneys active; pulse as before reported; œdema about the face and scalp on the decline; wound still discharging freely—condition healthy. One of the pins, having cut through, was removed, and the adhesive strips renewed. Cold applications continued.

March 1st.—Case progressed favorably until the afternoon, when another chill came on, more formidable in character than any of the preceding. Surface became more or less livid—extremities cold, with all the concomitants of a severe paroxysm. The discharge from the wound ceased; the "tumor" depressed where the bone were absent; pulse thread-like; eyes dull and sunken, with more or less difficulty of respiration; breathing hurried and irregular.

Revulsions were resorted to, together with stimulants—sinapisms to spine and extremities, champagne, dry heat, milk punch, etc.; reaction was established, and followed by fever, with more or less incoherence. The fever was kept within bounds by the free use of ice internally and externally, and passed away as usual with free cutaneous action, after the turn of the night.

March 2d.—Condition this morning comparatively good. Bowels opened with seidlitz powder; continued pills, with champagne, fish diet, and use of ice-water dressing to head. During the evening the discharge from the wound was reëstablished—the head dressed again, so as to allow of free exit of matter. The next chill paroxysm was anticipated with free application of stimulating linaments to spine, sinapisms to extremities, dry heat, etc., by which means it was warded off, and the case continued to improve steadily, the pills being continued. No application was made to the head except the cold water, or ice-water dressing by means of folds of lint frequently changed, throughout the entire treatment of the case. The discharge continued freely, but gradually diminished after the ninth or tenth day. The remaining pins came away soon after the first was removed, and afterwards the lips of the wound were supported in apposition by adhesive strips frequently changed.

The wound discharged by two orifices: the one just above the superciliary ridge, which was the first to close; the other just at the upper portion, where the blunt edge of the axe had driven the fragment of internal plate deep into the brain substance, and so injured the soft parts, or external scalp, as to cause some sloughing. The discharge from this orifice did not entirely cease until about the twenty-eighth day after the reception of the injury.

The case made a complete recovery, without the least injury to the mental powers in any particular, so far as can be ascertained from the patient himself, his parents, his teachers, or his associates.

Some points in this case are of such interest that it may not be amiss to refer to them here in a brief way.

At the time of receiving the injury, this patient *lost over two fluid ounces of brain substance from the middle anterior portion of the left lobe of the cerebrum*—cut out by the axe or removed in a broken-down condition; how much more by suppuration, in the process of healing, it is impossible to say, but evidently a considerable portion, from the amount of matter discharged from the wound before it entirely healed. In dressing the wound, care was taken to preserve the periosteum—to replace the cerebral membranes carefully in position, and to *briny the periosteum in contact with the dura mater*, with the hope that they might ultimately reproduce a bony covering. This hope was stimulated by the researches and results of experiments of Mons. Ollier and others. These results have not as yet been attained, after the lapse of over three years and a half, or at least are far from complete. By measurement, however, the opening in the bony covering has diminished to some extent through the projection of the edges toward the centre of the opening. These deposits being chrystalline in the outset, according to M. Ollier, the development of hard bony tissue may be retarded by the cerebral pulsations preventing the hard formation except where the deposits are protected by the edges of the existing bone. This would appear to be the case in this instance, from the measurements indicating the slow approach of the edges.

During the progress of this case, and to some extent still observable, there was a very great *depression* of the tegumentary covering *during sleep*. This filled out to the *level* whilst the patient was awake, and very materially *protruded* during fever or mental excitement. This, to a greater or less extent, according to the amount of fever or excitement, or rest or mental repose, obtains still. During the existence of a chill, the depression was very great, and the like depression was and is still seen when deep sleep overcame the patient. A condition of stertorous breathing approaching asphyxia in sleep showed an excessive depression from want of blood in the cerebral vessels. Stimulants or mental excitement would restore the normal condition or run to the other extreme.

It is well known to the profession that in all, or nearly all, cases of severe injury of the brain (cerebrum), the mind becomes in some way impaired, either temporarily or permanently. Some lose their consciousness, others their memory. With some, the past is obliterated, and after recovery, they have to learn their lessons anew; whilst others have lost their knowledge of figures, or some other of their faculties; and with most, their disposition is very materially changed. In this case, none of these results have followed. I have carefully examined him in every conceivable way, directly and indirectly, and cannot detect that in any particular he has lost or changed. Resuming his studies at school, (and he had an unusually bright intellect), he had retained what he had formerly learned; fond of music, of mathematics, history and the languages, he had retained what he had learned of these. At the time of receiving the injury, he had been, for twelve or eighteen months, giving more or less attention to telegraphing—a business requiring, as is well known, a very acute sense of time. After the injury, his instructor informed me that he had lost none of his powers in this respect, and that his sense of time was as acute as ever.

Not desiring to enter further, here, into the discussion of the many points of interest—physiological, pathological, psychological, or phrenological—presented in this case, it is respectfully submitted. This hurriedly-written history is compiled from notes taken during the progress of the case, presenting only the facts. Its future will be watched with much interest, both as regards the osteo-genetic powers of the periosteum and dura mater under the pulsating motion of the cerebral arteries, and as a case where a large amount of brain substance has been broken up and removed *without producing the least perceptible mental disturbance.*

